# Pangenome analysis of *Shewanella xiamenensis* revealed important genetic traits concerning genetic diversity, pathogenicity and antibiotic resistance

**DOI:** 10.1186/s12864-024-10146-z

**Published:** 2024-02-27

**Authors:** Haichen Wang, Fengjun Xia, Yubing Xia, Jun Li, Yongmei Hu, Yating Deng, Mingxiang Zou

**Affiliations:** 1 National Clinical Research Center for Geriatric Disorders, Xiangya Hospital, Central South University, Changsha, 41008 Hunan Province People’s Republic of China; 2 Department of Clinical Laboratory, Xiangya Hospital, Central South University, Changsha, Hunan Province People’s Republic of China

**Keywords:** *Shewanella xiamenensis*, Pan-genome analysis, Antibiotic resistance genes, Mobile genetic elements, Virulence genes

## Abstract

**Background:**

*Shewanella xiamenensis*, widely distributed in natural environments, has long been considered as opportunistic pathogen. Recently, significant changes in the resistance spectrum have been observed in *S. xiamenensis*, due to acquired antibiotic resistance genes. Therefore, a pan-genome analysis was conducted to illuminate the genomic changes in *S. xiamenensis*.

**Results:**

Phylogenetic analysis revealed three major clusters and three singletons, among which close relationship between several strains was discovered, regardless of their host and niches. The “open” genomes with diversity of accessory and strain-specific genomes took advantage towards diversity environments. The purifying selection pressure was the main force on genome evolution, especially in conservative genes. Only 53 gene families were under positive selection pressure. Phenotypic resistance analysis revealed 21 strains were classified as multi-drug resistance (MDR). Ten types of antibiotic resistance genes and two heavy metal resistance operons were discovered in *S. xiamenensis*. Mobile genetic elements and horizontal gene transfer increased genome diversity and were closely related to MDR strains. *S. xiamenensis* carried a variety of virulence genes and macromolecular secretion systems, indicating their important roles in pathogenicity and adaptability. Type IV secretion system was discovered in 15 genomes with various sequence structures, indicating it was originated from different donors through horizontal gene transfer.

**Conclusions:**

This study provided with a detailed insight into the changes in the pan-genome of *S. xiamenensis*, highlighting its capability to acquire new mobile genetic elements and resistance genes for its adaptation to environment and pathogenicity to human and animals.

**Supplementary Information:**

The online version contains supplementary material available at 10.1186/s12864-024-10146-z.

## Introduction

*Shewanella xiamenensis*, a member belonging to *Shewanella* genus, is a motile gram-negative and facultative anaerobic bacterium. *S. xiamenensis* was first identified from coastal area of Xiamen, China in 2010 [1]. *S. xiamenensis* is widely distributed in nature environment, mainly in ocean and coastal areas [2]. Recently, *S. xiamenensis* has been continually reported as an important opportunistic pathogen [3, 4]. A series of clinical manifestations, including skin and soft tissue infections, wound infections, cellulitis, bloodstream infections by *S. xiamenensis* have been reported. Animal infection by *S. xiamenensis* has been also reported [5]. Risk factors for *S. xiamenensis* infection may include open wounds, exposure to marine environments, and immunocompromised status. However, the pathogenic mechanisms and virulence genes have not been fully recognized among *S. xiamenensis*.

*S. xiamenensis* has been identified as an important carrier of antibiotic resistance genes, as it is considered as the source of *bla*_OXA−48_ [6]. Although multiple studies focused on *Shewanella* genus, the strains used were mostly isolated before 2019 [7]. At present, significant changes in the antibiotic resistance of *S. xiamenensis* have been unraveled. Recently, *S. xiamenensis* carrying *bla*_NDM−1_ or *tet*(X4) have been reported in China and Vietnam, respectively [8, 9]. The resistance against last-line antibiotics like carbapenems and tigecycline indicated that the gene pool of *S. xiamenensis* has significantly expanded, making it become a reservoir of antibiotic resistance genes in aquatic environment. However, only limited studies suggested that inserting sequences (ISs) may enhance the genomic diversity of *S. xiamenensis* and a few resistance plasmids have been sequenced among *Shewanella* spp. strains [10]. So, further study on the changes in *S. xiamenensis* genome is still needed.

Pan-genome analysis can help to quickly understand the basic genomic characteristics and functional diversity of *S. xiamenensis*. Therefore, a pan-genome construction on 50 *S. xiamenensis* genomes was performed to analyze the genomic diversity, mobile genetic elements, virulence genes and horizontal gene transfer (HGT) events, thus providing fundamental description focusing on the genome changes in *S. xiamenensis*.

## Materials and methods

### Genome collection and quality control

Two carbapenem-resistant *S. xiamenensis* were collected from wastewater samples in Xiangya Hospital. The details of isolation and sequencing were recorded in Supplemental Materials.

Genomes from NCBI Assembly database with keyword ‘Shewanella’ were downloaded on April 29, 2023. Considering the possible mis-assigned taxonomic identification, the average nucleotide identity (ANI) values of all genomes against *S. xiamenensis* reference strain HD6416 (GenBank no. GCA_024971755) were calculated by pyani (version 0.2.12) [11]. ANI values equal to or higher than 0.95 were preliminary considered as the same species [12, 13]. The misidentified genomes were further validated by calculating the amino acid identity (AAI), tetra-nucleotide signature (TETRA) and *in silico* DNA-DNA hybridization (DDH) values with CompareM (https://github.com/dparks1134/CompareM), pyani and the Genome-to-Genome Distance Calculator 3.0, respectively [11, 14]. Only under the conditions of ANI > 95%, AAI > 95%, and TETRA > 0.99 simultaneously, two genomes are considered as same genomic species.

The genomes passed quality control were introduced for pan-genome analysis. The completeness and contamination were analyzed by CheckM (version 1.0.11) [15]. The genomes of *S. putrefaciens* (GenBank no. GCA_025402875) and *S. algae* (GenBank no. GCA_009730655) were used to root the tree in phylogenetic analysis.

### Pan-genome construction and functional annotation

The genome sequences were annotated by Prokka (version 1.14.6) [16]. The orthologous gene families were identified by OrthoFinder 2 (version 2.5.4) with default parameters (DIAMOND method) [17]. The core, accessory and strain-specific genomes were identified according to the distribution of gene families among the genomes. The rarefaction curves were generated by a power-law regression based on Heaps’ law. Heap’s law uses values related to genome and pan-genome size as predictor and outcome, and power-law model use values related to genome size and the number of newly added gene clusters [18, 19]. The curve was visualized with Origin 2023 in Allometric1 model. The functions of gene families were annotated by eggNOG-mapper software (version 2.1.9) [20].

### Phylogenetic analysis

The core-genome phylogeny tree was built from single-copy orthologous sequences. The protein sequences in each single-copy gene family were aligned with MAFFT (version 7.508) [21]. The aligned protein sequences were back translated to nucleotide alignment with PAL2NAL (version 14.1) and concatenated [22]. The phylogeny was built by IQ-TREE 2 from core nucleotide alignments with ModelFinder Plus module [23]. The GTR + F + I + I + R5 was identified as the best model for phylogeny construction. ClonalFrameML (version 1.12) was employed to identify recombination and rescale branch lengths [24].

The pan-genome tree was built based on a binary presence/absence matrix representing the distribution of gene families among *S. xiamenensis* genomes. Manhattan distance was calculated to measure the evolutionary relationship and neighbor-joining tree was constructed with MEGA 11.0.

### Pressure selection analysis

The selection pressure at codon level was evaluated by calculating the ratio of non-synonymous rate to synonymous rate (dN/dS). The protein sequences in each gene family of core and accessory genomes were aligned by MAFFT and back translated to nucleotide alignment with PAL2NAL. The Fast Unconstrained Bayesian Approximation (FUBAR) of HYPHY (version 2.5.50) was employed to calculate the non-synonymous and synonymous replacement rates at each locus of a protein-encoding sequence [25].

### Comparative genomic analysis

The genome islands were annotated by IslandViewer 4 [26]. The prophages were identified by the PHAge search tool – Enhanced Release (PHASTER) [27]. Only hits with scores higher than 70 (intact and questionable prophages) were included in our study. The ISs were detected by ISfinder and VRprofile 2 [28, 29]. The results of ISfinder were classified according to previous work [30]. CRISPRCasFinder (version 4.3.2) and CRISPRCasTyper (version 1.8.0) were employed to detect Clustered Repetitively Interspaced Palindromic Repeat (CRISPR) arrays and Cas proteins [31, 32]. The antibiotic resistance genes and phenotypes were predicted by ResFinder 4.0. Bacteria that are non-susceptible to one or more agents in at least three categories, are identified as multi-drug resistant (MDR) [33]. The heavy metal resistance genes were identified by aligning the protein sequences against the BacMet2 database [34]. The virulence genes were identified by aligning the protein sequences against the Pathogen Host Interactions database (PHI-base 5.0) with DIAMOND BLASTP [35]. The macromolecular secretion systems were predicted by MacSyfinder (version 2.0) with default parameters [36]. The type 4 secretion system were verified by SecReT4 and oriTfinder [37, 38].

### Identification of potential horizontal genes and plasmid analysis

HGTector (version 2.0b3) was employed to identify potential horizontal transferred genes in *S. xiamenensis* strains [39]. The software automatically chose *Shewanella* (rank: genus; taxon ID: 22) and *Alteromonadales* (rank: order; taxon ID: 135,622) as self-group and close-group, respectively.

## Results

### Available genome sequences from public database for *S. xiamenensis*

Considering the misidentification of *S. xiamenensis* by traditional methods, we firstly checked the taxonomic classification of *Shewanella* spp. genomes. A total of 593 genome sequences were downloaded from NCBI Assembly database with keyword ‘Shewanella’. The ANI values of 592 genomes against *S. xiamenensis* HD6416 strain ranged between 81.60% and 99.99% (Supplemental Table S1). The ANI values shared by 47 genomes were higher than 95.00%, thus preliminarily determined as *S. xiamenensis*. The ANI values of other genomes were all less than 90.04% and excluded from this study.

Among the 47 genomes, 13 genomes were mis-identified as *Shewanella* spp. or *S. oneidensis*. The AAI and TETRA values of the 13 mis-identified strains against *S. xiamenensis* HD6416 strain were all higher than 97.52% and 0.9974, respectively. The DDH values against *S. xiamenensis* HD6416 strain ranged between 70.90% and 87.00%, which were higher than the recommended threshold level for species circumscription (70.00%). The ANI, AAI, TETRA, and DDH values all supported that the 13 strains should be re-identified as *S. xiamenensis* (Supplemental Table S2).

Therefore, a total of 50 genome sequences were selected for pan-genome analysis, including ten genomes involved in previous study [7]. The AAI and TETRA values of 49 genomes against *S. xiamenensis* reference strain HD6416 were all high than 97.48% and 0.99, respectively (Supplemental Table S3 and S4).

The *S. xiamenensis* strains were isolated from various samples, including water, lake sediment, soil, hospital wastewater and clinical sources, indicating its excellent adaptability to different environments (Supplemental Table S5). The genome size ranged between 4.423 Mb (S3C505, GCA_021209245) to 5.520 Mb (LC6, GCA_006385735). The genome completeness was greater than 92.08% and the contamination was less than 2.81%. The average number of coding sequence is 4,315, with a range between 3,933 (GCA_012490605) to 4,996 (GCA_006385735). The GC content of the *S. xiamenensis* genomes is 46.26 ± 0.119%, indicating a minor variation in GC content between *S. xiamenensis* strains.

### Pan-genome and phylogenetic analysis of *S. xiamenensis*

**Fig. 1:**
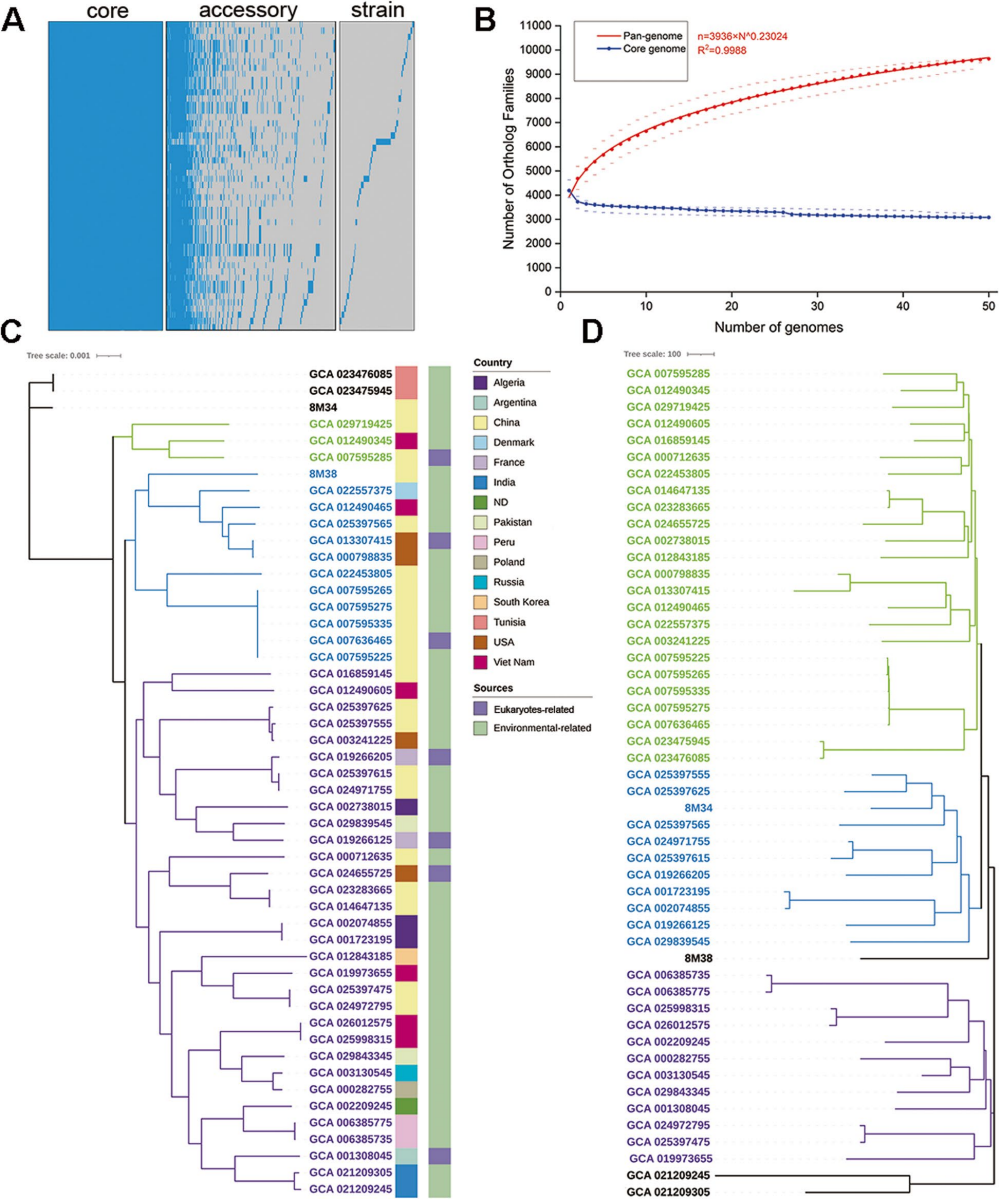
Pan-genome analysis of *S. xiamenensis*. **A** Presence/absence matrix of the gene families identified in *S. xiamenensis*. The pan-genome is subdivided into core, accessory and strain-specific genomes according to the distribution of gene families. **B** The accumulative curve for the genomes of *S. xiamenensis*. The curve represented the size of pan and core genomes as the number of genomes increased. **C** The single-copy gene tree generated by the core nucleotide alignments. The strains were clustered into three major groups (marked as green, blue and purple, respectively) and three singletons (marked as black). The first colored row at the end of branches represented the country where the strains were isolated. The second colored row represented the sources. **D** The pan-genome tree based on the binary presence/absence matrix of each gene families. The strains were clustered into three major groups (marked as green, blue and purple, respectively) and three singletons (marked as black)

In total, 7,643 orthologous gene families were identified, accounting for 99.1% of all genes. The pan-genome consisted of 9,636 gene families, and 3,080 (31.96%), 4,563 (47.35%) and 1,993 (20.69%) genes were classified into core, accessory and strain-specific genomes, respectively (Fig. 1A, Supplemental Table S6). Among core genome, 2,724 single-copy gene families were identified. The numbers of strain-specific genes differed significantly in 50 genomes, ranging from 1 to 394 (GCA_021209245) genes. The pan-genome accumulation curve constructed with Heap’s law model showed the expansion tendency for *S. xiamenensis* genome, with the exponent γ = 0.23 (Fig. 1B).

To investigate the genome diversity and relationship between strains, the single-copy gene tree and pan-genome tree based on presence/absence matrix of gene families were constructed (Fig. 1C and D). The phylogenic trees were classified into three clusters and three singletons. The close relationship between strains isolated from host and environment was observed (e.g., GCA_013307415 and GCA_000798835) in two trees. Two trees presented various degrees of difference in strain relationship. For example, GCA_003241225 (host-related, isolated in USA), GCA_025397625 (environmental-related, isolate in China) and GCA_025397555 (environmental-related, isolate in China) showed close relationship in single-copy gene tree. However, GCA_003241225 was no longer the sister in the pan-genome tree, while strain 8M34, which was a singleton in single-copy gene tree, showed weak evolutionary relationship to both strains. Furthermore, the pan-genome tree recognized much evolutionary distance than the single-copy gene tree (e.g., GCA_021209305 and GCA_021209245). In the rooted single-copy gene tree, the *S. xiamenensis* strains showed phylogenetically distant to other *Shewanella* spp. (Supplemental Figure S1).

### Functional enrichment and selection pressure analysis of *S. xiamenensis*

A total of 6,460 gene families (67.04%) were annotated into 21 Cluster of Orthologous groups (COGs) of proteins functional categories (Supplemental Table S6). A large proportion of genes (2,868, 93.12%) in core genome were assigned into functional categories, while in accessory and strain-specific genomes, only 2,535 (55.56%) and 1,057 (53.04%) genes were annotated into provisional functions, respectively (Supplemental Figure S2A). The core genome was significantly enriched into basic vital function including C (energy production and conversion), E (amino acid transport and metabolism), F (nucleotide transport and metabolism), H (coenzyme transport and metabolism) and J (translation, ribosomal structure and biogenesis) (Fish’s exact test, *P* < 0.001, 0.001, 0.001, < 0.001 and < 0.001, respectively). The accessory genome was enriched in function including K (transcription), L (replication, recombination and repair), N (cell motility) and S (function unknown) (Fish’s exact test, *P* = 0.014, < 0.001, 0.015 and 0.019, respectively). The strain-specific genome was significantly enriched in S (function unknown, *P* = 0.001, Fish’s exact test).

The dN/dS values of most gene families were less than 1, with a mean of 0.168 ± 0.224. Genes with certain COG catalogues were under different degrees of conservative selection pressure, and genes related to F (nucleotide transport and metabolism) and V (defense mechanisms) experienced strongest purifying pressure (Supplemental Figure S2B). The mean dN/dS value of core gene families (0.116 ± 0.120) was significantly lower than that of accessory gene families (0.178 ± 0.287; t test, *P* < 0.001). Furthermore, the core gene families for most COG categories had lower dN/dS values than that for the corresponding accessory gene families, except for F (nucleotide transport and metabolism), H (coenzyme transport and metabolism), I (lipid transport and metabolism), N (cell motility), Q (secondary metabolites biosynthesis, transport and catabolism) and V (defense mechanisms) (Supplemental Figure S2C).

In totally, 53 gene families shared dN/dS values more than 1, including three core gene families and 50 accessory gene families (Supplemental Table S7). Among these, 43 gene families encoded hypothetical proteins and the rest 10 gene families encoded abhydrolase, AcpP (Acyl carrier protein), YafO toxin, cytochrome *cbb*_*3*_, OprD, Transposase, YbfB (MFS-type transporter), PfpI, fumarate reductase and DUF4145 (function unknown), respectively. Besides the gene families under positive selection pressure on the entire coding region, a total of 2,670 gene families contained codon sites which were subjected to different degree of positive selection (Supplemental Figure S2D). The gene families (2,338, 87.57%) were identified into COG categories and enriched to: E (amino acid transport and metabolism), N (cell motility), P (inorganic ion transport and metabolism) and T (signal transduction mechanisms) (Fish’s exact test, *P* = 0.022, < 0.001, 0.003 and 0.039, respectively).

### Phenotypic resistance profiles and antibiotic resistance gens of *S. xiamenensis*

**Fig. 2:**
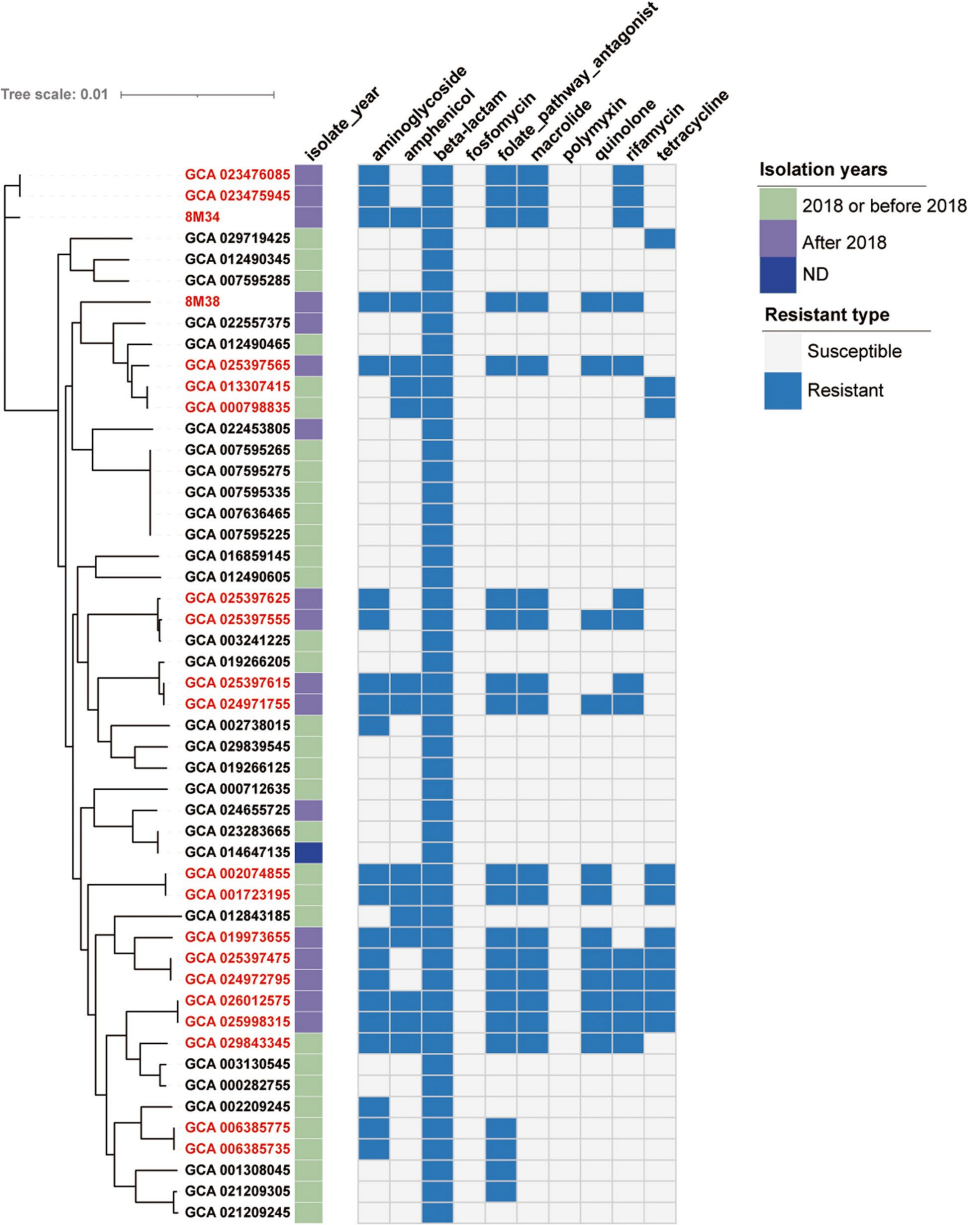
Isolation date and distribution of resistant spectrum for *S. xiamenensis*. The phylogenetic tree on the left was constructed by the single-copy genes among *S. xiamenensis*. The metadata on the right of the tree indicated the isolation data and resistant type. The blue squares represented that the strains were resistant to at least one agent in the categories of antibiotics. The strains marked in red represented that the strain was non-susceptible to at least one agent in at least three categories and were regarded as MDR.

Among the ten categories, 50, 21 and 21 strains were resistant to at least one agent of beta-lactam, aminoglycoside and folate pathway antagonist antibiotics, respectively (Fig. 2, Supplemental Table S8). All the *S. xiamenensis* were resistant to at least one kind of β-lactam antibiotic, including unknown beta-lactam (*n* = 32), amoxicillin (*n* = 31) and ampicillin (*n* = 31). For carbapenems, 31, 23 and 22 strains were resistant to imipenem, meropenem and ertapenem, respectively, due to the carriage of *bla*_NDM−1_ (*n* = 12) and *bla*_OXA−48_ variants (*n* = 19). Among aminoglycoside, 16 and 5 strains were resistant to gentamicin and amikacin, respectively. Three strains were predicted to be resistant to tigecycline. All *S. xiamenensis* strains were predicted susceptible to fosfomycin and polymyxin. However, the minimal inhibition concentration (MIC) value of 8M38 strain against fosfomycin was ≥ 1024 µg/ml (Supplemental Table S9).

A total of 21 strains were classified as MDR. Among the MDR strains, four strains were only resistant to three categories, including two strains resistant to amphenicol, beta-lactam and tetracycline, and two strains resistant to aminoglycoside, beta-lactam and folate pathway antagonist. The rest 17 strains were resistant to at least 5 categories, 28 to 37 kinds of antibiotics.

Ten types of antibiotic resistance genes were identified in *S. xiamenensis*, including trimethoprim, rifampicin, disinfectant, phenicol, beta-lactam, macrolide-lincosamide-streptogramin, sulphonamide, aminoglycoside, fluoroquinolone, tetracycline and amphenicol (Supplemental Table S10). Each genome carried 1 to 33 antibiotic resistance genes. The most prevalent antibiotic resistance genes were *bla*_OXA−48_ variants (*n* = 51), *sul1* (*n* = 48), *qacE* (*n* = 35), *mph(E)* (*n* = 15), *msr(E)* (*n* = 15) and *bla*_NDM−1_ (*n* = 12). Each *S. xiamenensis* genome carried at least one *bla*_OXA−48_ variants, except for GCA_023476085, in which *bla*_OXA−204_ and *bla*_OXA−538_ were identified. The *bla*_OXA−48_ variants mainly existed on chromosome and share a conserved genetic environment like *endA*-*sprT*-*orf*-*bla*_OXA−48_-*lysR*-*accA*, except for strain GCA_023476085, in which two ISSheS2 were found in the downstream of *bla*_OXA−48_. Among 18 genomes, one to five *sul1* gene were discovered. The 12 strains with *bla*_NDM−1_ were widely distributed among the phylogenetic trees. In addition, other important antibiotic resistance genes including *qnrVC6* (*n* = 12), *aac*(3)-IId (*n* = 11), *bla*_TEM−1B_ (*n* = 9), *ARR-3* (*n* = 9), *dfr27* (*n* = 9), *bla*_PER−1_ (*n* = 4), *tet(X4)* (*n* = 3), were identified in *S. xiamenensis* genomes. No homolog with acquired fosfomycin resistance genes was discovered.

Among 17 strains collected after 2018, 14 strains were MDR phenotype. Based on the single-copy gene tree, at least nine strains, which were designated into three clusters and exerted highly homologous, carried different antibiotic resistance genes, i.e., the later isolated strains have more resistance genes and exhibit MDR phenotype. For example, GCA_025397625 and GCA_025397555 acquired 11 and 17 antibiotic resistance genes, including *bla*_NDM−1_, respectively, when compared with their homology GCA_003241225, which only carried one *bla*_OXA48_ variants (Fig. 2).

### Heavy metal resistance genes carried among *S. xiamenensis*

Two heavy metal resistance operons, *mer* and copper homeostasis and silver resistance island, were identified in *S. xiamenensis* genomes (Supplemental Table S10). The mercury resistance *mer* operon was discovered in 21 genomes, in which 12 strains carried two *mer* operons. Among the 21 strains with *mer* operon, 19 were MDR phenotype. The copper homeostasis and silver resistance island in five *S. xiamenensis* strains consisted of *sil* operon (*silSRCFBAP*) and part of *pco* operon (*pcoDCBA*).

### Virulence genes and macromolecular secretion systems in *S. xiamenensis* genome

In total, 99 virulence genes have been identified among 50 genomes (Supplemental Figure S3). Among these, 57 (57.58%) virulence genes were presented in every *S. xiamenensis* genome. Most *S. xiamenensis* strains contained virulence genes for biofilm formation (*luxS*), capsular polysaccharide synthesis (*magA*), stress response (*rpoS*, *sodB*), protease (*lon*), Type III secretion system (*iscR*), pathogenicity island (*purA*), iron transporter (*fur*) and multi-kind regulators. The remaining 42 virulence genes distributed sporadically in *S. xiamenensis* genomes. On average, each genome carries 67.5 ± 2.32 virulence genes. A majority of these virulence genes were classified as “reduced-virulent” (64, 64.65%) for their mutant phenotype (Supplemental Table S11). We found 13 virulence genes associated with human, three of which were classified as hyper-virulent, and can lead to hemorrhagic colitis, skin infection, urinary tract infection, and listeriosis. In addition, 62 virulence genes were related to animals, and the potential hosts included rats (*n* = 35), roundworm (*n* = 6) and greater wax moth (*n* = 4). The remaining 24 virulence genes were associated with plants, mainly inducing soft rot, blackleg disease, leaf spot and fire blight.

**Fig. 3:**
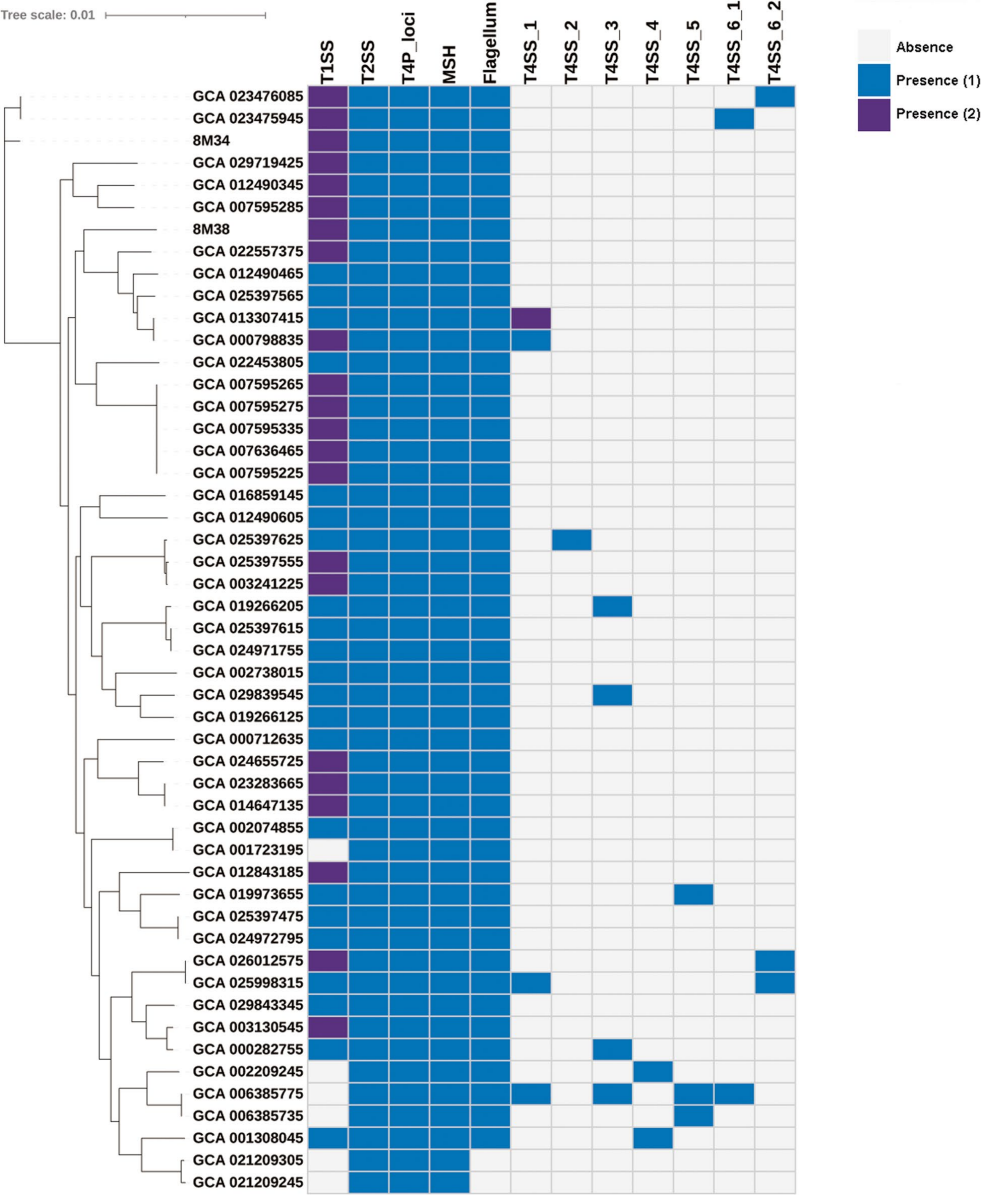
Heatmap representing the distribution of macromolecular secretion systems in *S. xiamenensis*. The blue and purple squares represented that the strains carried one or two secretion system of the corresponding type, respectively, and grey square represented absence. The phylogenetic relationship in the left was generated by the core nucleotide alignments of the single-copy genes

**Fig. 4:**
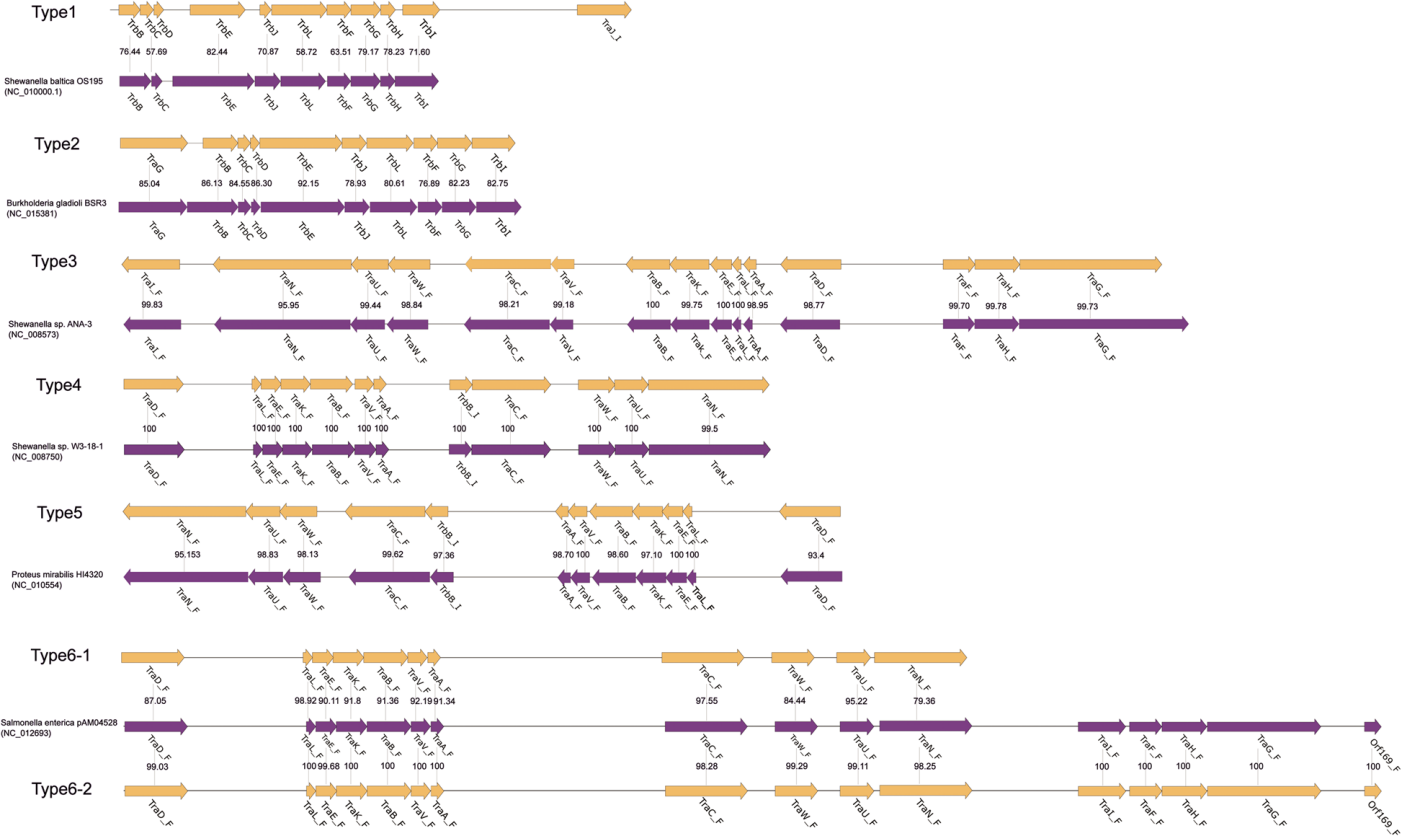
The different genetic structures of type 4 secretion system identified in *S. xiamenensis*. Two major types, six subtypes, were shown in the figure. The genes identified in *S. xiamenensis* were marked in yellow, and the genes identified in database were marked in purple. The homologous genes were linked by grey lines and the numbers represented the percentages of protein identities of homologous genes

For macromolecular secretion systems, T2SS, T4P loci and mannose sensitive hemagglutinin (MSH) were distributed in every genome (Fig. 3). Flagellum and T1SS were presented in 48 and 44 genomes, respectively. No T6SS was predicted. Diverse T4SS were discovered in in *S. xiamenensis* genomes, consisting of six types. For type6, two subtypes were identified, with a difference for subtype6-1 which lacked *traI*-*traF*-*traH*-*traG*-*orf169* sequence. T4SS distributed sporadically in 15 genomes (Fig. 4). According to the sequence similarity, T4SS were identified to originate from *Shewanella baltica* (Type1), *Burkholderia gladioli* (Type2), *Shewanella* sp. (Type3), *Acidovorax* sp. (Type4), *Proteus mirabilis* (Type5), *Salmonella enterica* (Type6-1 and 6 − 2), respectively. Three carried multiple T4SS. For example, two type1 T4SS were identified in strain GCA_013307415, while in strain GCA_006385775, four different T4SS were discovered, originated from diversity donor species.

### Genetic plasticity and genomic evolution mediated by genetic elements

**Fig. 5:**
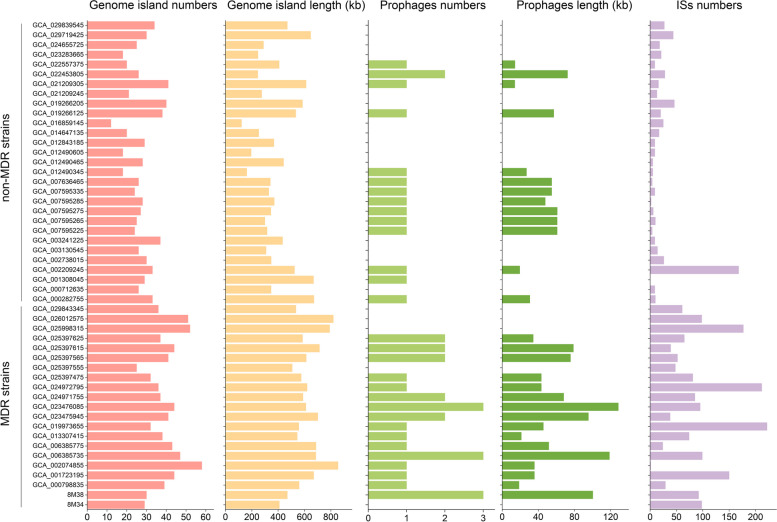
Distribution of mobile genetic elements (genome islands, prophages and ISs) in *S. xiamenensis*, including the number and length of genome islands, the number and length of prophages and the number of ISs (from left to right). The strains were clustered according to their resistance phenotypes

A total of 2,423 ISs were identified in the 50 genome of *S. xiamenensis*, belonging to 170 types (Fig. 5). Among these, IS10A (*n* = 182) was the most prevalent, followed by TnAs3 (*n* = 153), and TnAs2 (*n* = 89). In all genomes, the numbers of 55 ISs types were above 10, accounting for 2,095 (86.46%), which constituted the majority of ISs in *S. xiamenensis*. These ISs belonged to 15 families and mainly originated from *Shewanella* (*n* = 27), *Salmonella* (*n* = 6) and *Aeromonas* (*n* = 5). On average, each genome carried 48.46 ± 55.08 ISs. The distribution of ISs in *S. xiamenensis* genome was diversity. The MDR phenotype carries significantly more ISs (1-222 ISs; t test, *P* < 0.001) than sensitive strains (0-168 ISs). However, the non-MDR strain FDAARGOS_354 (GCA_002209245) carried 168 ISs with only one antibiotic resistance gene *bla*_OXA−199_.

The average numbers of genome islands and prophages in each genome were 32.44 ± 9.65 (485.27 ± 178.35 kb in size) and 0.84 ± 0.86 (31.41 ± 34.79 kb in size), respectively. Genome islands consisted of 9.75 ± 3.29% sequences in each genome. For MDR strains, the average length of genome islands and prophages were 623.99 ± 110.27 kb (t test, *P* < 0.001) and 47.41 ± 39.20 kb (t test, *P* = 0.005) in size, respectively, which were longer than that in non-MDR strains (genome islands: 384.82 ± 148.37 kb; prophages: 19.83 ± 25.58 kb). In total, 79 integrons were identified in 31 genomes of *S. xiamenensis* using VRprofile. The average number of integrons in MDR strains (2.71 ± 2.29; t test, *P* < 0.001) was significantly more than that in non-MDR strains (0.76 ± 1.04).

In *S. xiamenensis*, a total of 27 CRISPR/Cas systems were identified in 21 genomes of *S. xiamenensis*. The most common is I-F, followed by IIIB, I-E, and I-C. Five strains carried two CRISPR/Cas systems. Various spacers were found among different CRISPR/Cas systems, with numbers of 87–121, 46–145, 1-154 and 21–37 for I-C, I-E, I-F and III-B, respectively.

### Horizontal gene transfer in *S. xiamenensis*

**Fig. 6:**
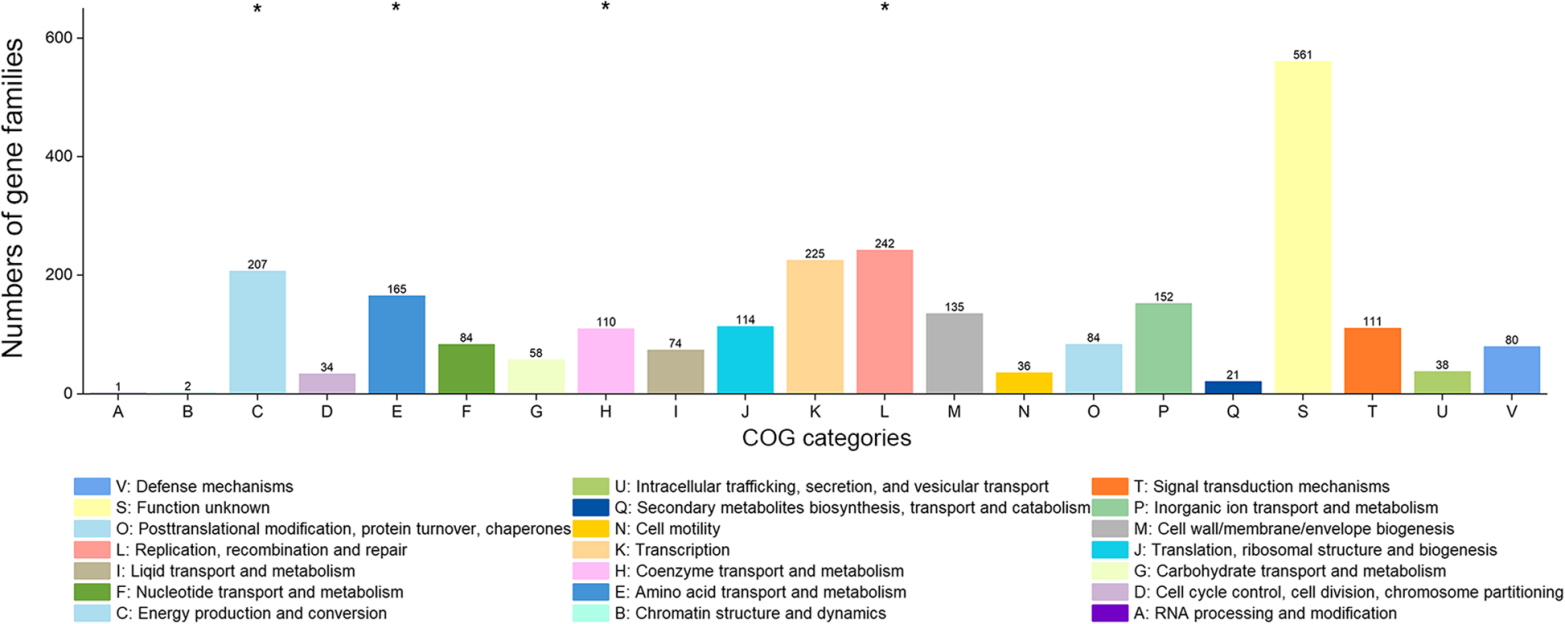
Distribution of horizontal transferred genes in each COG functional categories. Asterisk (*) represented that genes in the corresponding category were enriched in the transferred genes (Fish’s exact test, *P* < 0.05)

In *S. xiamenensis*, we used HGTector to identify potential horizontal transferred genes. In total, 64,631 potential horizontally transferred genes were identified in 50 genomes, belonging to 2,579 gene families, accounting for 26.76% of the pan-genome. Among them, 1,153, 1,127 and 299 genes families belonged to core, accessory and strain-specific genomes, respectively. On average, each genome of *S. xiamenensis* contains 1,292.56 ± 72.04 transferred genes. The MDR strains (1,355.00 ± 48.58) carried significantly more horizontal transferred genes than non-MDR ones (1,247.34 ± 48.68; t test, *P* < 0.001). The potential donors of the transferred genes mainly included members of *Vibronales*, *Aeromonadales* and *Burkholderiales* families. Functional annotation confirmed that genes related to C (energy production and conversion), E (amino acid transport and metabolism), H (coenzyme transport and metabolism) and L (lipid transport and metabolism) (Fish’s exact test, *P* = 0.005, < 0.001, 0.004, and 0.005, respectively) were enriched in transferred genes (Fig. 6).

## Discussion

In this article, we provided a detailed description of pan-genome characteristics of *S. xiamenensis*. Although there are previous works focusing on *Shewanella* spp., our results demonstrated a clear shift in antibiotic resistance for *S. xiamenensis* recently [7, 40]. Mobile genetic elements and macromolecular secretion systems may aid in this process. Furthermore, *S. xiamenensis* carries many virulence genes and is potential pathogenic towards human and animals.

In the pan-genome matrix of *S. xiamenensis*, a total of 9,636 gene families were identified, which was comparable to previous work [40]. However, the relatively small number of strain-specific genes was discovered, probably because the close relationship between *S. xiamenensis* strains, as demonstrated in phylogenetic analysis (Fig. 1). The accumulation curve showed that pan-genome still expanded with the increase of new genomes even including 9,635 genes, indicating an “open” genome for *S. xiamenensis*. The phylogenetic analysis revealed three major cluster and three singletons. Among these, several strains showed high homology, regardless of their niches and sources, indicating that *S. xiamenensis* in the environment is also potential pathogenic to human. As shown in Fig. 1, a divergence between two trees was observed. The discordant topology reflected different phylogenetic distances of *S. xiamenensis* and maybe due to the distribution of accessory and strain-specific genomes, which played an important role in adapting the environment. The pan-genome and phylogenetic analysis demonstrated the genomic diversity in *S. xiamenensis* strains.

A large proportion of core genome was annotated into provisional function, compared with accessory and strain-specific genomes, which reflected the specific evolutionary traits of the *S. xiamenensis* genomes. Genes related to housekeeping function, including energy, metabolism and cell components were enriched in core genome, indicating the need for *S. xiamenensis* to survive under extreme and hypo-trophic environment such as marine niche. The accessory genome was enriched in transcription, replication and cell motility, which were related to adaptation and influenced by different habitats. Most gene families were under purifying selection, as dN/dS values were less than 1. Genes related to nucleotide metabolism and defense mechanisms experienced the strongest purifying selection in all the COG categories, while the core genome shared lower dN/dS values, compared with accessory and strain-specific genomes. The lower dN/dS values indicated that these genes were under constraint conservation in the evolution process, prone to remain the basic functions and played an important role in key movements. In total, 53 gene families were proven to be under positive selection, and most encoded hypothetical proteins. The acyl carrier protein AcpP is essential for fatty acid synthesis and can interact with multiple functional proteins [41]. Toxin YafO is encoded by the yafN-yafO antitoxin-toxin operon and can inhibit protein synthesis [42]. Cytochrome c is an enzyme to exchange electrons during oxidation processes and plays an important role in the complex oxidative respiration chain in *S. xiamenensis*. The *pfpI* gene, a member of the ThiJ/DJ-1/PfpI family, has been proven to confer protection against stress [43]. Furthermore, 2,670 gene families contained codon sites under diversifying positive selection and enriched in function including transport, inorganic ion, cell motility and signal, indicating that these functional genes may involve in the adaptation to diverse habitats. An average of 1,292 genes per genome were predicted to be horizontal transferred and the main donors included *Vibronales*, *Aeromonadales* and *Burkholderiales* families. The closely relationship between these donors and *S. xiamenensis* may accelerate the HGT events. The transferred genes mainly enriched in amino acid, coenzyme and lipid metabolism, and energy production, which can expand the gene pool of *S. xiamenensis* and enhance the adaptability to environment. The functional analysis provided basic information to understand the evolutionary characteristics of *S. xiamenensis*.

*S. xiamenensis* has been continually reported as opportunistic pathogen, leading to skin and soft tissue infections, wound infections, cellulitis and bloodstream infections. A various of virulence genes were identified in *S. xiamenensis* genomes, which further supported these previous reports [3, 4]. The risk of infection by *S. xiamenensis* cannot be underestimated, especially for immunocompromised patients. Macromolecular secretion systems are equipment implanted on cell membrane and secrete effector factors, involving in key biology process, including nutrition acquisition, environment adaptation, inter-communication and virulence gene expression (Fig. 3). T2SS, T4P loci and MSH were presented in all genomes while flagellum and T1SS distributed in most genomes, which were closely related to various bacterial activities, including adhesion, motility, chemotaxis, biofilm formation and secretion of virulence factors, indicating these secretion systems played an important role in the adaptation and pathogenicity of *S. xiamenensis* [44–46]. T4SS was also prevalent in *S. xiamenensis* genomes (15/50, 30%). The diverse structures of T4SS and complex donor strains indicated that these T4SS were horizontal transferred. Furthermore, three strains with more than one T4SS were found, a sign for multiple HGT events or a combination of transfer and duplication may occur. T4SS can secrete effector molecules and mediate conjugation and transformation, playing an important role in horizontal gene transfer and improving survival and pathogenicity [47]. Taken together, *S. xiamenensis* have multiple virulence factors, pathogenic to potential host including humans and animals and can lead to a variety of diseases.

The more worrying situation is the antibiotic resistance of *S. xiamenensis*. Based on ResFinder, 21 strains were classified as MDR phenotype and a majority (14/21, 66.7%) were isolated after 2018, indicating the necessity to consider the resistance phenotype when treating *S. xiamenensis* infection. Two important antibiotic resistance genes, *bla*_NDM−1_ and *tet*(X4), were identified among *S. xiamenensis*, conferring high-level resistance to carbapenem and tigecycline. Another antibiotic resistance gene, *bla*_OXA−48_ variants, were present in each genome, in line with the speculation on the origin of this variants, which have spread to *Enterobacterales* and conferred low-level resistance to carbapenems and associated with carbapenem treatment failure [48, 49]. Interestingly, at least nine strains, which were grouped into three cluster with highly homologous in the single-copy gene tree, showed different carriage of antibiotic resistance genes. The homology among core genome and difference in antibiotic resistance may suggest that these strains shared similar genetic backgrounds but acquired different genetic elements, including antibiotic resistance genes, ISs and prophages. The genetic distances for these strains in the pan-genome tree further reflected the divergences in presence/absence of gene families. Fosfomycin has been considered as an effective alternative for carbapenem-resistant pathogens. However, although sensitive phenotype for fosfomycin was predicted, our antimicrobial susceptibility test confirmed an MIC value above 1024 µg/ml. No *fos* genes have been discovered, so it is speculated that the resistance may be caused by the mutations of fosfomycin target MurA, fosfomycin transport system (GlpT and UhpT) and its regulatory genes. Similar resistance towards fosfomycin of *Shewanella* spp. has been reported, suggesting that the utilization of fosfomycin on the treatment of carbapenem-resistant *S. xiamenensis* should be fully considered [10].

As for heavy metal resistance genes, the mercury resistance *mer* operon and silver/copper resistance island were discovered in *S. xiamenensis* genomes. The *mer* operon was highly correlated with MDR phenotype, indicating that these heavy metal and antibiotic resistance genes can locate in the same plasmid or mobile genetic element and co-transfer through HGT [50]. In addition, the *sil* operon (*silSRCFBAP*) was discovered in 5 genomes. The silver resistance *sil* (*silSRCFBAP*) and copper resistance *pco* (*pcoESRDCBAFG*) operons often form gene clusters, named the copper homeostasis and silver resistance island [51]. Tn7 transposons usually surrounds the gene cluster, which plays an important role in the horizontal gene transfer of the operons [52]. However, in *Shewanella* spp., the structure of this island consisted of *silSRCFBAP* and *pcoDCBA*. The difference in genetic structure may indicate that this island was vertical transferred, rather than HGT. The carriage of these resistance genes demonstrated that *S. xiamenensis* has become an important reservoir and played an important role in the transmission of antibiotic and heavy metal resistance in aquatic environments.

Mobile genetic elements, including genome islands, prophage, ISs and integrons, are important vectors for DNA transfer, expanding the gene pool [53]. A total of 2,423 ISs were identified, with an average of 48.46 in each genome, which were significantly more than the average number of 4–12 ISs per genome reported before [7]. The origins of ISs were from *Shewanella*, *Salmonella* and *Aeromonas*, indicating that HGT between bacteria in aquatic environments may occur. Strains with MDR phenotype carried significantly more ISs than non-MDR ones, suggesting the close relationship between antibiotic resistance genes and ISs. But further study on the impact of ISs towards MDR is still needed, as one strain with 168 ISs and only one antibiotic resistance gene was discovered. As for prophage, genome islands and integrons, similar increase in numbers among MDR strains were also observed, suggesting that mobile genetic elements were important components for *S. xiamenensis*, contributing to the genetic diversity and HGT for antibiotic resistance. CRISPR and Cas protein are important defense mechanism for prokaryote against the invasion of genetic elements such as bacteriophages or plasmids [54].

## Conclusions

With an “open” genome, a great level of diversity was observed among *S. xiamenensis*, with the existence of accessory and strain-specific genomes, contributing to the adaptation to different niches. Purifying selection pressure was the main force in the evolution process. *S. xiamenensis* showed potentials to acquire important antibiotic resistance genes and mobile genetic elements, and exerted MDR phenotype, making this species a reservoir for the spread of antibiotic resistance. Taken together, our study provides the fundamental characteristics of *S. xiamenensis* pan-genomes, emphasizing the severe resistance and the potential spread of antibiotic resistance genes from *S. xiamenensis*.

### Supplementary Information

**Supplementary Material 1.**

**Supplementary Material 2.**

## Data Availability

The sequences generated during the current study were deposited in GenBank under the accession number of PRJNA992975.

## Abbreviations

ISs: Inserting sequences
HGT: Horizontal gene transfer
ANI: Average nucleotide identity
AAI: Amino acid identity
TETRA: Tetra-nucleotide signature
DDH: DNA-DNA hybridization
CRISPR: Clustered repetitively interspaced palindromic repeat
MDR: Multi-drug resistance
COG: Cluster of orthologous groups
MIC: Minimal inhibition concentration
MSH: Mannose sensitive hemagglutinin
