# Supplementary material for: Pangenome analysis of Shewanella xiamenensis revealed important genetic traits concerning genetic diversity, pathogenicity and antibiotic resistance
Source: BMC Genomics. 2024 Feb 27;25:216. doi: 10.1186/s12864-024-10146-z (PMC10898099; doi:10.1186/s12864-024-10146-z)
Supplement: Supplementary file 2 — Supplementary Material 2. [file 12864_2024_10146_MOESM2_ESM.docx]

**Supplemental materials：**

Pangenome analysis of *Shewanella xiamenensis* revealed important genetic traits concerning genetic diversity, pathogenicity and antibiotic resistance

Haichen Wang^1, 2^, Fengjun Xia^1, 2^, Yubing Xia^1,2^, Jun Li^1, 2^, Yongmei Hu^1, 2^, Yating Deng^1, 2^, Mingxiang Zou^1, 2*^

1 National Clinical Research Center for Geriatric Disorders, Xiangya Hospital, Central South University, Changsha, Hunan Province, People’s Republic of China

2 Department of Clinical Laboratory, Xiangya Hospital, Central South University, Changsha, Hunan Province, People’s Republic of China

*Correspondence:

Mingxiang Zou, PhD

Address: National Clinical Research Center for Geriatric Disorders, Xiangya hospital, Central South University, Changsha, 41008, Hunan Province, People’s Republic of China

Tel: +86 7384327440

Fax: +86 7384327440

E-mail: zoumingxiang@csu.edu.cn;

Experiments:

Carbapenem-resistant *S. xiamenensis* strains isolated from hospital wastewater

Two *S. xiamenensis* strains were isolated by agar screening method from raw wastewater in Xiangya Hospital at May 2022. The raw wastewater samples were collected and pooled on MAC agar (Oxoid, Hampshire, UK) supplemented with 100 μg/ml vancomycin (Sangon Bio, Shanghai, China) and 8 μg/ml meropenem (MeilunBio, Liaoning, China). After 24 h at 37 °C, the colonies were randomly chosen according to the morphology and identified by MicroflexTM MALDI-TOF MS system (Bruker Daltonik, Bremen, Germany). The strains were further validated as carbapenem resistant by broth microdilution method. The carbapenem resistance genes (*bla*_NDM-1_, *bla*_KPC-2_) were screened by PCR method.

Genome sequencing and De novo genome assembly

The genomic DNAs were extracted by SDS-based DNA extraction method and purified by Monarch^®^ Genomic DNA Purification Kit (New England Biolabs Inc., Ipswich, United States). The quality and quantity of DNA were examined by agarose gel electrophoresis, Nanodrop^TM^ One spectrophotometer and Qubit fluorometric instrument (ThermoFisher, Carlsbad CA, USA), respectively. The DNAs were sequenced with Oxford Nanopore PromethION platform.

The high-quality sequencing reads were assembled by unicycler (version 0.4.8). The taxonomic analysis for the sequences of the two strains were conducted by KmerFinder 3.2^1^.

Results

Basic characteristics of two carbapenem-resistant *S. xiamenensis* strains

From hospital wastewater, two strains of *S. xiamenensis* were isolated. Both strains were mis-identified as *S. putrefaciens* by MALDI-TOF MS system. PCR only detected carbapenem resistance gene *bla*_NDM-1_ in both strains. The antibiotic susceptibility results showed that both strains were resistant to gentamicin, ceftazidime, cefepime, ceftriaxone, ceftazidime/avibactam, trimethoprim/sulfamethoxazole, meropenem, imipenem and ertapenem. Strain 8M38 were further resistant to aztreonam, cefoperazone/sulbactam, and fosfomycin. The MIC values were listed in Supplemental Table S9. Filter mating experiment was unsuccessful for both strains, indicating that the plasmids were non-conjugative.

The basic sequencing data were listed in Supplemental Table S12. The ANI values of two strains against *S. putrefaciens* reference strain 4H (GenBank no. GCA_025402875.1) were 84.70% and 84.58%, respectively, indicating the misidentification for both strains. The strains were classified as *S. xiamenensis* or *Shewanella* sp. by KmerFinder-3.2. The ANI values of two strains against *S. xiamenensis* reference strain HD6416 (GenBank no. GCA_024971755) were 97.52% and 97.50%, respectively. These results indicated that bacterial classification based on whole genome information is superior in the identification of bacteria within the same genus over MALDI-TOF MS, which is based on proteins.


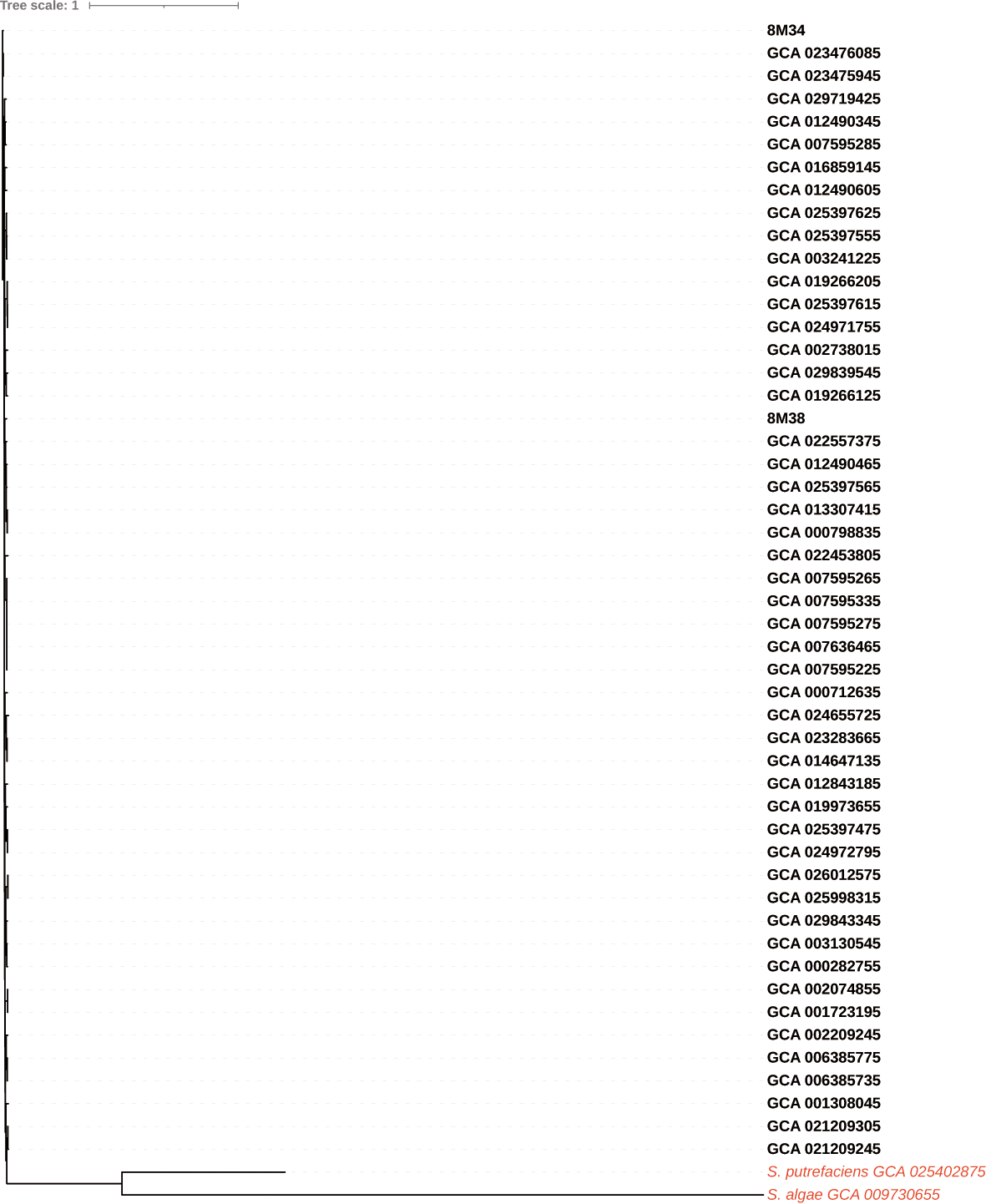
Figure S1 Rooted core genome tree based on single-copy gene families. The genomes of *S. putrefaciens* (GenBank no. GCA_025402875) and *S. algae* (GenBank no. GCA_009730655) were used to root the tree.


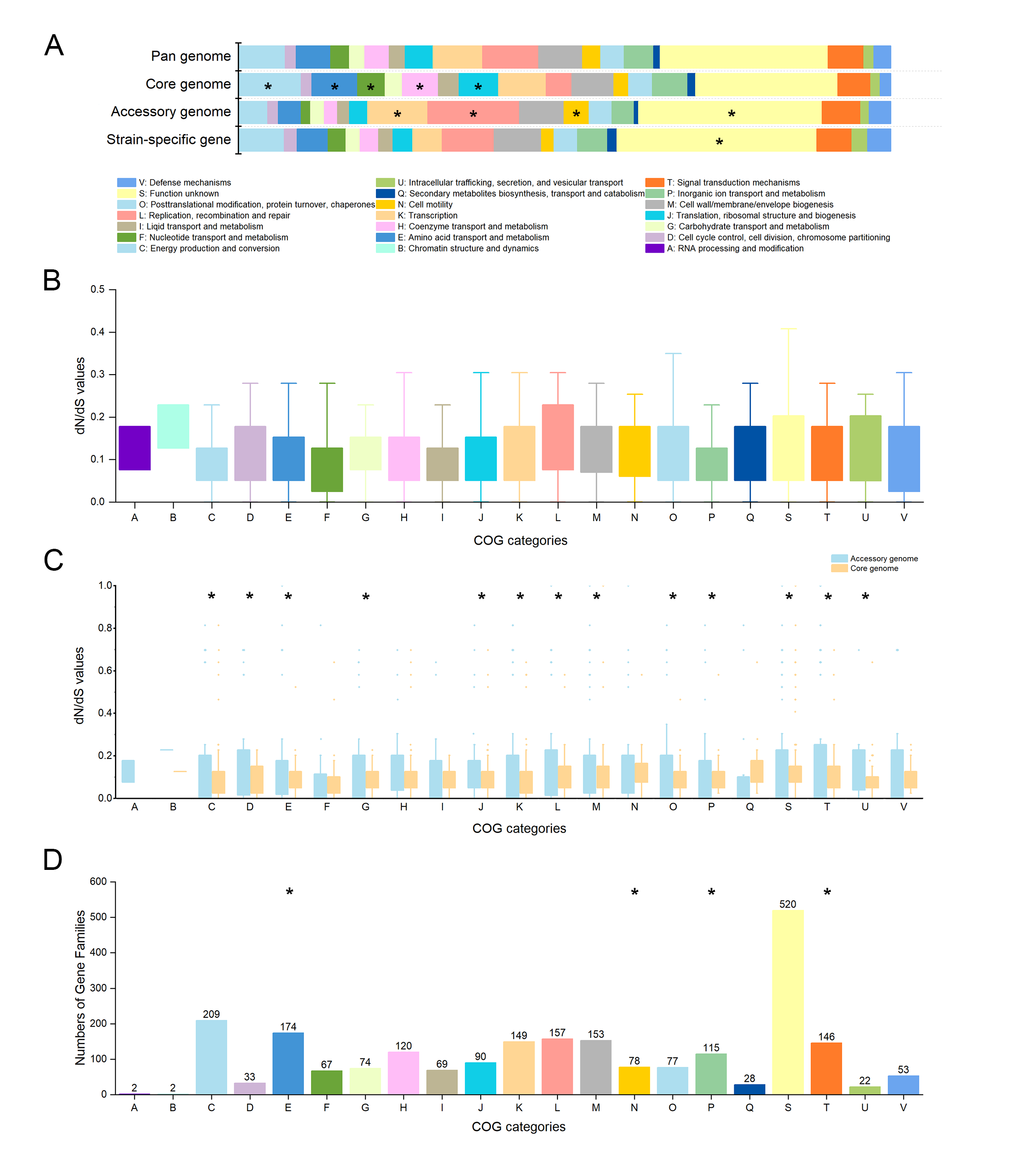
Figure S2 Function analysis of the *S. xiamenensis* pan-genome. (A) Distribution of COG categories for pan, core, accessory and strain-specific genomes, respectively. The length represents the proportion of each functional gene families in the corresponding genomes. Asterisk (*) represented that genes in the corresponding category were enriched in the corresponding genomes (Fish’s exact test, P < 0.05). (B) The ratio of non-synonymous rate (dN) to synonymous rate (dS) according to the COG categories. (C) dN/dS values for core and accessory genomes in each COG category, respectively. Asterisk (*) represented significant difference for dN/dS values in COG categories between core and accessory genomes (t test, P < 0.05) (D) Distribution of COG categories for gene families with codon sites under positive selection. Asterisk (*) represented that genes in the corresponding category were enriched (Fish’s exact test, P < 0.05).


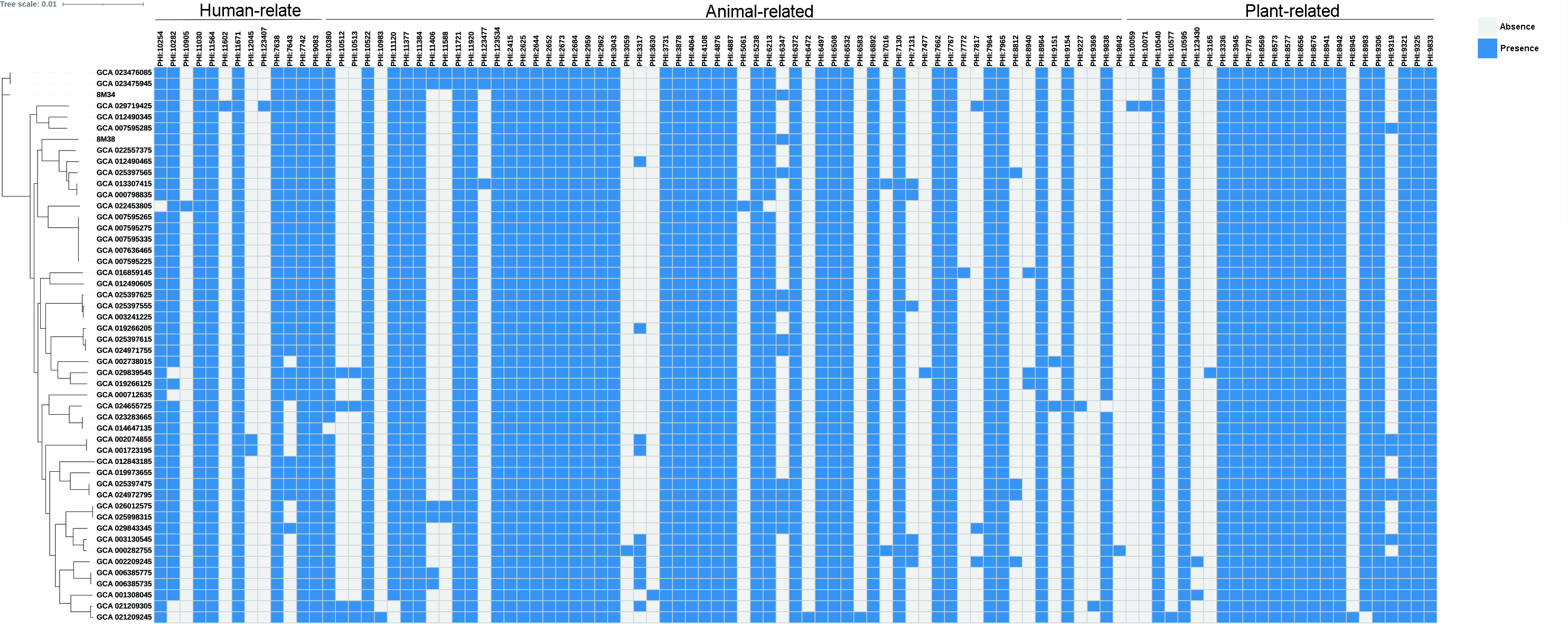


Figure S3 Heatmap representing the distribution of virulence genes among *S. xiamenensis* genomes. Blue square represents the presence of a gene, and grey square represents the absence. The phylogenetic relationship in the left was generated by the core nucleotide alignments of the single-copy genes.

References

1. Clausen P, Aarestrup FM, Lund O. Rapid and precise alignment of raw reads against redundant databases with KMA. *BMC Bioinformatics*. Aug 29 2018;19(1):307. doi:10.1186/s12859-018-2336-6
